# Pharmacological and Antioxidant Activities of *Rhus coriaria* L. (Sumac)

**DOI:** 10.3390/antiox10010073

**Published:** 2021-01-08

**Authors:** Halima Alsamri, Khawlah Athamneh, Gianfranco Pintus, Ali H. Eid, Rabah Iratni

**Affiliations:** 1 Department of Biology, College of Science, United Arab Emirates University, Al-Ain 15551, UAE; 200813902@uaeu.ac.ae (H.A.); khawlah.athamneh@ku.ac.ae (K.A.); 2 Department of Chemistry, College of Arts and Sciences, Khalifa University, Abu Dhabi 127788, UAE; 3 Department of Medical Laboratory Sciences, College of Health Sciences, University of Sharjah, Sharjah 27272, UAE; gpintus@sharjah.ac.ae; 4 Department of Biomedical Sciences, University of Sassari, Viale San Pietro 43, 07100 Sassari, Italy; 5 Department of Basic Medical Sciences, College of Medicine, QU Health, Qatar University, Doha 2713, Qatar; Ali.eid@qu.edu.qa; 6 Biomedical and Pharmaceutical Research Unit, QU Health, Qatar University, Doha 2713, Qatar

**Keywords:** *Rhus coriaria*, phytochemical, antioxidant, anticancer, antimicrobial

## Abstract

*Rhus coriaria* L. (Anacardiaceae), commonly known as sumac, is a commonly used spice, condiment, and flavoring agent, especially in the Mediterranean region. Owing to its bountiful beneficial values, sumac has been used in traditional medicine for the management and treatment of many ailments including hemorrhoids, wound healing, diarrhea, ulcer, and eye inflammation. This plant is rich in various classes of phytochemicals including flavonoids, tannins, polyphenolic compounds, organic acids, and many others. By virtue of its bioactive, *Rhus coriaria* possesses powerful antioxidant capacities that have ameliorative and therapeutic benefits for many common diseases including cardiovascular disease, diabetes, and cancer. This review describes the phytochemical properties of *R. coriaria* and then focuses on the potent antioxidant capacities of sumac. We then dissect the cellular and molecular mechanisms of sumac’s action in modulating many pathophysiological instigators. We show how accumulating evidence supports the antibacterial, antinociceptive, antidiabetic, cardioprotective, neuroprotective, and anticancer effects of this plant, especially that toxicity studies show that sumac is very safe to consume by humans and has little toxicity. Taken together, the findings we summarize here support the utilization of this plant as an attractive target for drug discovery.

## 1. Introduction

**Figure 1:**
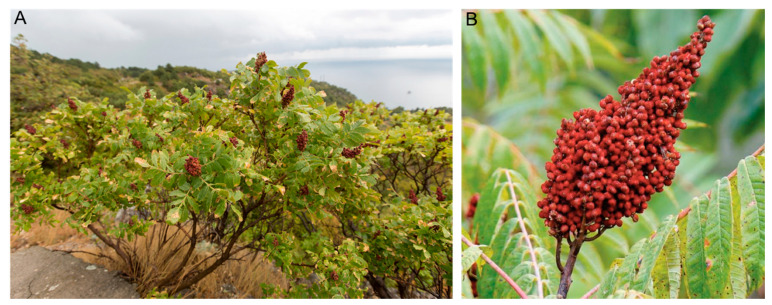
(**A**) *Rhus coriaria* plant and (**B**) *Rhus coriaria* fruit.

*Rhus coriaria* L., commonly known as sumac, is a Mediterranean plant that belongs to the Anacardiaceae family and is traditionally used as a spice and flavoring agent [1]. It grows as a shrub with a height range of 3–4 m and has pinnate leaves arranged in pairs of 6 or 8 small leaflets, with a cluster of white flowers at terminal inflorescences (Figure 1A). The fruits are spherical and become reddish drupe when ripe (Figure 1B) [2]. Dried fruits, reduced to a dark red powder, with an acidic and astringent taste is often used as spice in several Mediterranean and Middle Eastern countries such as Lebanon, Syria, Jordan, Turkey, and Iran [3]. For example, powdered sumac is often directly added to salad or offered along with minced meat to add lemony taste. In some countries like Lebanon and Syria, powdered sumac is sometimes used in the composition of zaatar, a mixture of herbs and spices in infusion or as a replacement for capers and red pepper. At the industrial level, Sumac leaves and bark, which contain large amounts of tannins, were used for centuries in tanning fine leather [4]. In addition to its use as a culinary herb and tanning agent, *Rhus coriaria* has been used in Middle Eastern and South Asian countries, for thousands of years, as a traditional medicine for the treatment of several diseases including cancer [5,6,7]. Sumac fruits were used in folks medicine to treat several illnesses that include liver disease [8], diarrhea [8,9,10], urinary system issues [8], and ulcers [11]. In addition, the powdered fruits were also used to stimulate perspiration and reduce cholesterol [10]. The many therapeutic effects of *Rhus coriaria* could be attributed to its various biological properties such as antioxidant, anti-inflammatory, hypoglycemic, hypolipidemic activities [2]. As of today, over 200 phytochemicals were isolated from *Rhus coriaria* and these include organic acids, phenolic acids, phenolic compounds conjugated with malic acid derivatives, flavonoids, isoflavonoids, hydrolysable tannins, anthocyanins, terpenoids, and other compounds such as butein, iridoid, and coumarin derivatives. This review aims to provide a comprehensive overview of the phytochemical and pharmacological studies published on *Rhus coriaria*.

## 2. General Composition and Minerals and Vitamins Contents of *Rhus coriaria*

The overall composition of the dried sumac fruit is mainly composed of moisture (6–11.8%), essential oil content (1.0%), protein (2.3–2.6%), fiber (14.6–22.15%), ash (1.5–2.66%), and water-soluble extract (63.8%) and fatty oil (17.4%) [12]. The mineral composition of sumac fruits, determined using inductively coupled plasma atomic emission spectrometer (ICP-AES), showed that K, Ca, Mg, P, Fe, Na, Zn, Mn, Cu, and Al are the predominant elements [13,14,15]. It is worth mentioning that mineral contents were found to be affected by environmental factors and the geographic locations where sumac fruits were collected [16]. As for the vitamin content, sumac fruit contained thiamine, riboflavin, pyridoxine, cyanocobalamin, nicotinamide, biotin, and ascorbic acid [16].

## 3. Phytochemical Constituents of *Rhus coriaria* Extract

*Rhus coriaria* is rather rich in many phytochemical compounds. One of the earliest studies carried out in 1896 identified myricetin and gallic acid as components of the leave extract [14]. Since then, many other components were identified in different parts of sumac (Table 1). More recently, a most comprehensive study investigating the phytochemical composition of the fruit extract identified 211 phytoconstituents including tannins, (iso)flavonoids, terpenoids, anthocyanins, and many others. Importantly, 180 of the 211 identified compounds were characterized for the first time in *Rhus coriaria* fruits [17].

## 4. Phytochemical Constituents of *Rhus coriaria* Essential Oil

Essential oils are natural oils extracted from different parts of the plant. They are complex mixtures of low molecular weight compounds that can be obtained by different means of extraction such as steam distillation, hydro-distillation, or solvent extraction. They are usually stored in oil ducts, resin ducts, glands, or trichomes of the plants. Essential oils have been used as raw materials in many fields, including perfumes, cosmetics, and foods. Moreover, interest in the use of essential oils in the treatment of many health-related conditions has been steadily increasing over the past years [15,33].

Essential oils extracted from *Rhus coriaria* have received much attention lately. In the early 1990s, different research teams extracted essential oils from different parts of sumac—especially its fruits—in an attempt to identify the chemical constituents of the oils using hydro-distillation as an extraction approach. It is worth noting that the yield of the oil using this extraction method is usually around 0.1% as *Rhus coriaria* is considered to be an essential oil-poor plant [34,35]. However, a more recent study showed that microwave-assisted extraction considerably improved the yield of sumac oil to ~13.5% [36].

Despite many studies on the non-volatile components of *Rhus coriaria*, little is known about the volatile composition. In 2018, the volatile profile of sumac fruit from three different origins (Palestine, Jordan, and Egypt) was reported. Also, the volatile profile of its cold tea and post roasting preparation was determined. 74 volatile components belonging to alcohols, aromatics, esters, ethers, furan/aldehyde, hydrocarbons, ketones, monoterpenes, oxides, and sesquiterpene hydrocarbons were identified. In fresh *Rhus coriaria* fruit, sesquiterpenes hydrocarbons accounted for the major volatile class, while furan/aldehydes were the major classes in the roasted fruits. Moreover, the volatile profile changed according to the geographic location where the fruit was collected. Egyptian sumac, for instance, showed more difference among its tea or roasted profiles as compared to Palestinian and Jordanian sumac, which both showed similar profiles [5]. Another study investigated the volatile and sensory flavor profiles of *Rhus coriaria* fruit obtained from the southeastern region of Turkey [37]. This study showed that malic acid, present in the volatile fraction, accounted for the sour taste of *Rhus coriaria* fruit. Other compounds, namely β-caryophyllene (spicy, woody), cembrene (woody), and caryophyllene oxide (spicy), are believed to contribute to the flavor of *Rhus coriaria* fruit [37]. Essential oil composition and the volatile profile of *Rhus coriaria* are illustrated in Table 2.

## 5. Antioxidant Activities of *Rhus coriaria*

Oxidative stress is caused by the imbalance between production and elimination of the reactive oxygen species (ROS) [43]. Phytochemicals and especially phenolic compounds are known as secondary metabolites and are known to possess potent antioxidant effects. Recent epidemiological studies show that consumption of plant materials with antioxidant activity may decrease the risk of several diseases [44]. Owing to its potent antioxidant capacities, summarized in Table 3, *Rhus coriaria* may be useful in the management or treatment of several pathological disorders, such as skin injuries [45], myopathies [46], overweight, and obesity [47].

Antioxidant agents have been used in the treatment of skin disorders for their ability to efficiently reduce the damage induced by sunlight [48,49,50]. The most dangerous components of the solar radiation are ultraviolet rays A (UV-A) and B (UV-B). While both rays can damage epidermal cells, UV-A can penetrate deeply into the dermal layers [51]. The photoprotective effects of the macerated ethanol extract of *Rhus coriaria* L. fruit on microvascular endothelial cells (HMEC-1), a model of the skin microvascular endothelium cells, have been investigated [45]. Importantly, a protective role of *Rhus coriaria* L. extract against UV-A-induced damage was reported. Indeed, *Rhus coriaria* extract not only reduced the level of UV-A-induced ROS production, but also significantly blocked the formation of DNA lesion in cells exposed to medium UV-A doses [45]. In contrast, when cells were exposed to higher and more damaging doses of UV-A, *Rhus coriaria* extract promoted cell cycle arrest and apoptosis [45]. However, the molecular mechanism through which the extract exerts its antioxidative and genoprotective effects remain unclear and thus warrant further investigations.

Oxidative stress, mainly via ROS, is associated with both physiology and pathology of skeletal muscle. Indeed, ROS is intimately linked to muscle fatigue [43]. This suggests that protecting myoblasts from oxidative stress can enhance muscle function. Contextually, in human myoblasts and zebrafish embryos subjected to oxidative stress by hydrogen peroxide, *Rhus coriaria* extract evoked a very powerful antioxidative effect, where it inhibited or slowed down the progress of skeletal muscle atrophy. This appears to be due to the extract’s ability to suppress oxidative stress through superoxide dismutase 2 and catalase [46]. Thus, the use of natural antioxidants in treating muscular pathologies or delaying disease development is promising.

A documented role for ROS and lipase inhibition in obesity is existent. Lipases present in the digestive system are important in the hydrolysis of glycerides into free fatty acids and glycerol. The most important of these lipases is the pancreatic lipase (PL) which plays an important role in converting the triglycerides found in ingested oils into monoglycerides and free fatty acids [52]. Hence the inhibition of this enzyme reduces fat absorption and hence represents an excellent strategy to prevent and treat obesity. Also, increasing evidence strongly suggests that elevated oxidative stress is critically involved in obesity and in the pathogenesis of obesity-associated metabolic syndromes [53]. The antiobesity and antioxidants activity of *Rhus coriaria* was first evaluated by Jamous et al., They showed that *Rhus coriaria* leaves and fruit epicarp exhibited a potent inhibition of LP activity in vitro [47]. In addition, sumac extract also showed a strong antioxidant and scavenging activity measured by the α, α-Diphenyl-β-picrylhydrazyl (DDPPH) scavenging activity assay [28,47]. Another recent study by Taskin et al. revealed that methanolic extract of sumac leaves exhibited a potent antioxidant and scavenging activity in DPPH, FRAP, CUPRAC, and ABTS assays [54]. Interestingly, a recent study by Heydari et al. showed that sumac fruit supplementation led to significant weight loss, decrease of waist circumference, and body mass index in obese patients [55]. Hence, *Rhus coriaria*—through its antioxidant and pancreatic lipase inhibition—represent a valuable source for potential natural compounds that could be beneficial in the fight against overweight or obesity.

Liver is one of the many victims of oxidative stress, evident by increased associated between ROS levels and liver damage. Importantly, in isolated rat hepatocytes, aqueous extract of *Rhus coriaria* protects against cumene hydroperoxide (CHP)-induced oxidative stress [56]. Results show that *Rhus coriaria* extract protected hepatocytes against several oxidative instigators such as glutathione depletion, lysosomal membrane oxidative damage, ROS generation, lipid peroxidation, cellular proteolysis, or mitochondrial membrane potential decrease [56]. Interestingly, gallic acid, one of the similar hepatoprotective effect, suggestive of a potential partial contribution of gallic acid to sumac’s hepatoprotection [56].

The DNA protective activity of *Rhus coriaria* was examined by Chakraborty and his group. Using single cell gel electrophoresis assay with freshly prepared human lymphocytes, the authors showed that *Rhus coriaria* fruit extract was able to effectively prevent H_2_O_2_- and (±)-anti-benzo[*a*]pyrene-7,8-dihydro-diol-9,10- epoxide (BPDE)-induced DNA-damage, and to reduce the endogenous formation of oxidized purines and pyrimidines induced by formamidopyrimidine glycosylase (FPG) and endonuclease III (ENDO III) enzymes, respectively [57,58]. This protective effect was confirmed in an in vivo study. Indeed, *Rhus coriaria* extract was able to significantly reduce DNA damage in all organs (colon, liver and lung) of irradiated rats [57].

*Rhus coriaria* seems to exert its protective effects by increasing the activities of detoxifying enzymes of the overall glutathione S-transferase (GST) and the two isozymes (GST-*α* and GST-*π*) in plasma of human subject treated with sumac extract [57]. In addition, *Rhus coriaria* might also reduce DNA-damage through a direct ROS scavenging activity [57]. Gallic acid, a major constituent in sumac fruit, was also shown to reduce H_2_O_2_-induced DNA damage in human lymphocytes in level comparable to sumac [57]. Hence, gallic acid, although maybe not solely, could account for the genoprotective effect of the sumac extract.

## 6. Pharmacological and Biological Activities of *Rhus coriaria*

Over the past recent years, several studies demonstrated the wide range of pharmacological and biological activities of the different parts of *Rhus coriaria*. These activities, which are summarized in Table 3, include antioxidant, antimicrobial, antidiabetic, cardioprotective and antidyslipidemia, antinociceptive, neuroprotective, dental protection, and anticancer effects.

### 6.1. Antibacterial Activities

Nowadays, there is an explosive increase in bacterial resistance to many antibiotics, making this issue a serious threat to humans [129]. One way to control and limit this resistance is identifying new antibacterial agents, ideally with novel mechanisms. The use of plants as potential sources for new drugs is an attractive route and is indeed receiving more attention. To this end, plants are rich with ethnomedicinal constitutes that play a distinguished role in the maintenance of human health against several diseases [130]. In this context, Zhaleh et al. recently assessed the antibacterial activities of the essential oil of *Rhus coriaria* on several bacterial strains. They showed that sumac’s essential oil efficiently prevents the growth of *Pseudomonas aeruginosa*, *Escherichia coli*, and *Staphylococcus aureus* or *Bacillus subtilis* with concentrations of 2, 3, or 15 mg/mL respectively [62].

Aqueous or ethanol extract of *Rhus coriaria* were also tested for antibacterial activities [59,63]. In this context, ethanolic extract of sumac fruits showed robust concentration-dependent antimicrobial activity with a broad spectrum for all tested bacterial strains, which included both Gram-positive and Gram-negative bacteria. *Salmonella enterica* and *Staphylococcus aureus* showed the most sensitivity toward sumac fruit’s ethanolic extract, with a minimum inhibitory concentration (MIC) of <0.78%. This is addition to a similar inhibitory capacity of ethanolic extracts of ripe and unripe fruits of sumac against Gram-positive and Gram-negative bacteria strains such as *Bacillus cereus*, *Klebsiella pneumoniae*, *Escherichia coli*, *Proteus vulgaris*, *Shigella dysentariae*, *Pseudomonas aeruginosa*, *Staphylococcus aureus*, *Staphylococcus epidermidis*, *Streptococcus pyogenes*, *Yersinia enterocolitica*, and *Enterococcus faecalis* [63]. These results support the traditional use of *Rhus coriaria* as disinfectant and deserves further studies toward the isolation of novel antimicrobial molecules that could be employed to treat microbial infections.

*Streptococcus mutans* is a well-known facultative anaerobic bacterium responsible for pathogenesis of dental caries and tooth decay. Kacergius et al. showed that methanolic extract of *Rhus coriaria* significantly inhibits the growth of *Streptococcus mutans* [64]. A greater inhibitory effect was obtained with methyl gallate, a major constituent of *Rhus coriaria* extract [64]. In another study, aqueous extract of sumac demonstrated a concentration-dependent growth inhibition of five common oral bacteria namely, *Streptococcus mutans*, *Streptococcus sanguinis*, *Streptococcus sobrinus*, *Streptococcus salivarius*, and *Enterococcus faecalis* [69]. Moreover, on orthodontic wire, the plant extract was also able to significantly reduce bacterial biofilm formation by *Streptococcus mutans*, *Streptococcus sobrinus*, *Streptococcus salivarius*, and *Enterococcus faecalis* [69]. It is noteworthy that *Rhus coriaria* extract did not show significant effect against the growth of beneficial bacteria [67]. All these data argue in favor of *Rhus coriaria* as a potential source of novel compounds that possess antibiofilm activity, and which could be used for oral health.

### 6.2. Antifungal Activities

Fungi are also important pathogens that infect humans, animals and even plants. As such, they can cause several diseases and lead to crop losses. Anthracnose is a disease caused by a number of the fungal pathogens such as *Colletotrichum acutatum* which frequently attack temperate plants, causing damage to both mature and immature fruits [131]. The efficiency of *Rhus coriaria* towards the control of tomato anthracnose, caused by *Colletotrichum acutatum* in tomato plants and fruits, was tested. Interestingly, aqueous extract of sumac’s fruits elicited significant antifungal activity against tomato anthracnose caused by *Colletotrichum acutatum*, suggesting that *Rhus coriaria* can be a cost-effective and ecofriendly replacement to chemical fungicides in the management of tomato anthracnose disease [46].

### 6.3. Antidiabetic Activities

Diabetes mellitus is one of the most common metabolic disorders, characterized by insulin malfunction and hyperglycemia due to inadequate insulin secretion, or both of them [132]. In 2019, 463 million adults worldwide were estimated to have diabetes mellitus, and these figures are expected to rise to 700 million by 2045 [133]. The effects of *Rhus coriaria* on type II diabetes was studied by examining serum glycemic status, apolipoprotein (apo) B, apoA-I and total antioxidant capacity (TAC) in type II diabetic patients. Type II patients consuming a powder of *Rhus coriaria* (3.0 g, daily for 3 months) showed a significant decrease in the levels of serum glucose, HbA1c, ApoB, and ApoA-I and an increase in the total antioxidant capacity [78]. Furthermore, administration of *Rhus coriaria* hydroalcoholic seed extract significantly decreased the level of glucose and cholesterol in nicotinamide-streptozotocin-induced type II diabetic male mice [73]. Furthermore, LDL-cholesterol level decreased while the level of leptin significantly increased in those mice when treated with a dose of 300 mg/kg of the hydroalcoholic extract [73].

In male diabetic patients, one of many complications is infertility, with around 90% of patients suffering from this rather life-changing effect [134]. It appears that testicular weight, sperm count and viability, serum luteinizing hormone (LH), follicle-stimulating hormone (FSH), and testosterone levels are significantly lower in the diabetic mice [135]. Interestingly, treatment of these male diabetic mice with 400 mg/kg of the hydroalcoholic extract of *Rhus coriaria* seeds led to a significant improvement in these parameters [135]. These studies show that sumac seeds, in addition to favorably regulating glycemia, also reduces its complications such as the risk of infertility.

### 6.4. Cardioprotective and Antidyslipidemia Activities

Cardiovascular disease (CVD) is the leading cause of death worldwide. CVD is a result of longstanding risk factors, such as high blood pressure, an atherogenic diet, and dyslipidemia [136,137]. Herbal drugs and alternative medicine have been taken into consideration in managing cardiovascular risk factors. *Rhus coriaria* is known to be rich in bioactive compounds that can improve cardiovascular health. Indeed, in patients with dyslipidemia, a triple-blind randomized placebo-controlled crossover trial reported improvement in endothelial vasodilator function, including flow-mediated dilation (FMD), after consumption of a daily dose of 500 mg of *Rhus coriaria* fruits for 4 weeks [103]. Furthermore, significant reduction in the systolic and diastolic blood pressure, total serum cholesterol and low-density lipoprotein cholesterol (LDL-C), non-high-density lipoprotein cholesterol (non-HDL-C), and body mass index (BMI) was observed in the *Rhus coriaria* treated group compared to the placebo one [103]. A meta-analysis study revealed that *Rhus coriaria* exerts a positive effect on different indices of the lipid profile including increasing Apo A-I and HDL, decreasing Apo B, Apo B/ Apo A1 ratio, total cholesterol, LDL, and triglyceride [105]. Another study showed that methanolic extract of *Rhus coriaria* leaves (RCLE) exerts a cardiovascular protective effect in isolated rabbit heart, which when perfused with various concentrations of RCLE for 20 min prior to ischemia, was dose-dependently protected against myocardial injury caused by ischemia–reperfusion [107]. Indeed, RCLE significantly reduced the activity of two markers of myocardial damage, creatinine kinase, and lactate dehydrogenase. Mechanistically, RCLE seems to exert its cardioprotective effect through inhibiting TNF-α, activating endothelial nitric oxide synthase (eNOS) and cyclooxygenases (COX), and scavenging free radicals and ROS [107]. Our group recently reported that the ethanolic extract of *Rhus coriaria* fruit, dose-dependently relaxes rat isolated aorta. This effect is mediated through stimulation of multiple signaling cascades which include PI3-K/Akt, eNOS, nitric oxide (NO), soluble guanylate cycle (sGC), cGMP, protein kinase G (PKG), COX, adenylyl cyclase/cAMP and ATP- gated potassium channels [102]. In addition to our previous finding as an antihypertensive, ongoing experiments in our lab are also deciphering the potential effect of vasorelaxant activity of sumac on other blood vessel type and not only on the aorta. It appears from our preliminary results that Sumac fruit extract is cardiovascular protective as well (RI and AHE, personal communication). Altogether, these findings strongly support the favorable potential cardio- and vasculo-protective action of *Rhus coriaria* and opens the doors toward the advantageous use of folk medicine for amelioration of cardiovascular diseases such as atherosclerosis, aortic aneurysms, and hypertension.

### 6.5. Antinociceptive Activities

Pain is an unpleasant sensation associated with many ailments. It has always been a serious challenge in medicine to find effective pain medication with fewer side effects. Therefore, the focus toward the use of medicinal plants has been increasing in recent years. A study investigating the analgesic effects of *Rhus coriaria* hydroalcoholic leaf extract showed a significant reduction in writhing in Wistar rats pretreated with sumac extract [101]. This sumac-modulated reduction in writhing was concomitant with the increase in the tail-flick time and inhibition of both phases of the formalin test, suggestive of an antinociceptive effect of *Rhus coriaria*. Hence, these results allude to the notion that the pain-relieving effect of sumac may be mediated through both peripheral and central mechanisms [101]. Based on these findings, *Rhus coriaria* can be a potential source for the novel, natural, and safe compounds with an analgesic effect and thus deserves further investigation in clinical trials.

### 6.6. Neuroprotective Activities

Neurodegenerative diseases can be initiated by severe acute traumatic injuries, ischemia, hypoxia generating oxidative stress, and neuroinflammation which is strongly associated with one of Alzheimer’s diseases such as optic neuropathies [138]. Retinal degeneration is known to cause a visual impairment and blindness and it can occur when the blood supply to the retina is unable to meet the ocular metabolic needs [139,140]. Therefore, the need for neuroprotective medication or any therapeutic approaches that reverse, block, or slow down neuronal cell death in neurodegenerative diseases is urgently needed. A study investigating the neuroprotective effects of the ethanolic extract of *Rhus coriaria* (ERC) fruit against retinal degeneration in vitro was conducted in the rat retinal ganglion cell line RGC-5 [58]. Results showed that ERC significantly reduced serum-deprivation-induced cell death of RGC-5 cells. Also, ERC was found to significantly abolish the reduction in the levels of GST and GSH induced by serum deprivation [58]. These findings highlight a potential neuroprotective activity of *Rhus coriaria* against retinal degeneration and justify its use in folk medicine. The limitation of this study is the use of a single retinal cell line. Further neural cell lines should be tested, and animal studies should be undertaken to further confirm the neuroprotective activity of sumac fruit.

### 6.7. Dental Protection Activities

Enzymatic degradation of the dentin matrices results in the loss of stability of resin–dentin bonds built by contemporary adhesives [141]. Matrix metalloproteinases (MMPs) are endogenous proteases known to be responsible for the turnover of collagen-based tissues [142]. Even though MMPs are inactive in mineralized dentin, the application of acidic monomers or etching acids can uncover and activate these proteases causing a progressive loss, over time, of collagen from the composite layers of tooth [143]. A study by Seseogullari-Dirihan et al. evaluated the MMP activity of demineralized dentin matrix following pretreatment by various collagen crosslinkers including *Rhus coriaria* fruit extract [144]. This extract significantly reduced total dentin MMP activity compared to control [144]. Moreover, zymogram analysis of dentin powder treated with *Rhus coriaria* extract confirmed a decrease in MMP-2 and MMP-9 activities. Furthermore, multiplex bead analysis of extracts of *Rhus coriaria*-treated dentin showed a reduction in the release of MMP-8, MMP-2, and MMP-9 [144]. Thus, *Rhus coriaria* may serve as a source for new MMP inhibitors that could be useful in the prevention or treatment of dental disease.

### 6.8. Anticancer Activities

**Figure 2:**
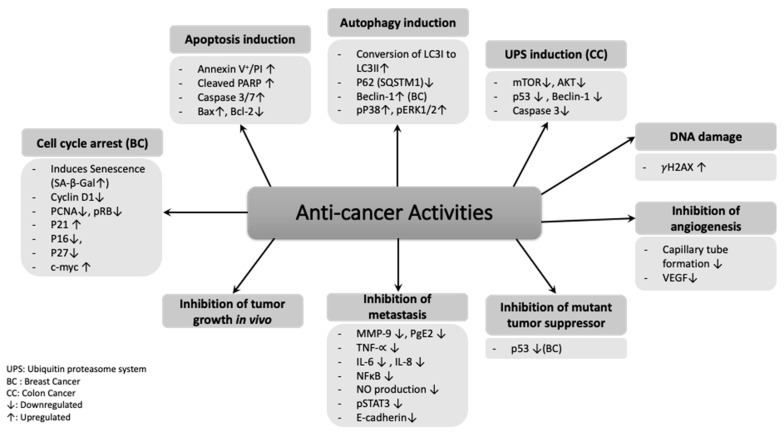
An overview on the molecular targets of *Rhus coriaria* against cancer.

Although great advancements in the treatment and control of cancer progression have been achieved, the undesired side effects that are accompanied with chemotherapeutic drugs can cause severe health problems. Therefore, natural therapies, such as using plant-derived bioactive compounds with less adverse side effects, gained much interest nowadays in cancer treatment and prevention [145,146]. *Rhus coriaria* is one of those plants with proven anticancer activities against various types of cancers. Figure 2 summarizes the effect of *Rhus coriaria* against different cancers.

#### 6.8.1. Breast Cancer

Our group was the first to explore the anticancer activity of *Rhus coriaria* in breast cancer. *Rhus coriaria* ethanolic extract (RCE) was found to decrease the cellular viability of various breast cancer cell lines (MDA-MB-231, MCF-7, and T47D) in a time- and concentration-dependent manner. In addition, RCE was shown to induce DNA damage, irreversible G1 arrest and senescence associated with the expression of senescence-associated β-galactosidase [121]. Analysis of cell cycle regulators revealed an RCE-induced upregulation of p21 and downregulation of cyclin D1, p27, PCNA, c-myc, and phospho-RB [121]. We also showed that RCE reduced cell viability through the activation of the type II programmed (autophagic) cell death. Mechanistically, RCE elicits its effect through autophagy-dependent activation of p38 and ERK1/2 signaling pathways. Indeed, inhibition of autophagy reduced cell death and blocked p38 and ERK1/2 phosphorylation [121]. In another study, we reported that RCE significantly inhibits the migration, fibronectin adhesion and invasion of the triple negative MDA-MB-231 breast cancer cells [119]. In addition, RCE efficiently inhibited angiogenesis by preventing tube formation of capillary like structures by HUVEC cells. In vivo, RCE led to the inhibition of tumor growth and metastasis of MDA-MB-231 in chick embryo model [119]. In 2018, silver nanoparticles coated with aqueous extract of *Rhus coriaria* (AgSu/NPs) reduced cellular viability and induced apoptotic cell death in MCF-7 breast cancer cells line [117]. Altogether, these findings identify *Rhus coriaria* as a promising chemopreventive and therapeutic candidate that modulate breast cancer growth and metastasis. Quercetin, one of the first flavonoid identified in the composition of *Rhus coriaria*, was shown to reduce cell viability, induce autophagy by inhibition of the AKT/mTOR pathway and triggers caspase-3 dependent apoptosis in various cancer cells [147]. Hence, it is legitimate to suggest that quercetin can contribute, maybe not solely, to the anticancer activity of *Rhus coriaria*.

#### 6.8.2. Colon Cancer

Our group was also the first to investigate the anticancer effect of *Rhus coriaria* ethanolic extract on two human colorectal cancer cell lines namely, HT-29 and Caco-2 [118]. RCE decreased cellular viability of the two cancer cell lines, inhibited the colony growth of HT-29 cells and slowed down HT-29 tumor growth in vivo using the mouse xenograft model [118]. Interestingly, in colon cancer, RCE activates both cellular programmed cell death (apoptosis and autophagy) pathways. Mechanistically, RCE exerts these effects through a stimulation of global protein ubiquitination and the ubiquitin proteasome system (UPS). As a consequence, component of the AKT/mTOR signaling pathway along with other proteins such as p53, Beclin-1, and caspase-3 were targeted to proteasomal degradation. The role of the UPS in RCE -mediated anti-colon cancer effect was further confirmed using inhibitors of proteasome activity. Indeed, MG132 significantly reduced RCE-induced apoptotic and autophagic cell death of colon cancer. Further investigations are underway to isolate the compound(s) modulating proteasome activity in colon cancer cells.

#### 6.8.3. Uterus, Cervix, and Retinoblastoma Cancer

The anticancer activity of *Rhus coriaria* ethanolic extract on uterus cervix cancer using HeLa cells was determined. Indeed, noncytotoxic concentrations of *Rhus coriaria* reduced migration of HeLa cells in the wound healing assay [31]. *Rhus coriaria* resin extract was also shown to cause cytotoxic and antiangiogenic effects against the retinoblastoma Y79 cell line [120]. Unfortunately, investigations undertaken to conclusively assess the anticancer activity of *Rhus coriaria* against uterus cervix and retinoblastoma cancer are still insufficient. As such, in vitro and in vivo studies are recommended to delineate the potential anticancer efficacy of this plant against these two forms of cancer.

### 6.9. Anti-Inflammatory Activities

It is well known that chronic inflammation is implicated in the development and progression of many chronic diseases such as autoimmune and neurodegenerative diseases, atherosclerosis, obesity, diabetes, cardiovascular diseases, and cancer. Chronic inflammation represents, nowadays, the most significant cause of death (>50%) worldwide [148]. Existing biomarkers, which include, but not limited to CRP, IL-1β, IL-6 and TNF-α, IL-8, IL-17, IL-12, IL-23, and CCL5, have been useful for demonstrating that inflammatory activity is involved in the pathogenesis of chronic diseases and mortality risk [148,149]. Cortisol and anti-inflammatory natural products were shown to provide protections on a number of chronic diseases. The anti-inflammatory activity of *Rhus coriaria* was reported by several groups. Momeni et al. were among the first to show a potential anti-inflammatory activity of *Rhus coriaria*. The authors demonstrated that sumac fruit alcoholic extract significantly reduced the level of mRNA of the pro-inflammatory cytokines IL-18 and IL-1β, in lipopolysaccharide-stimulated synoviocyte extracted from joint and fluid of limb of the 8-month-old healthy calf [122]. In another study, Gabr *et al*. showed that *Rhus coriaria* fruit extract accelerates the healing of induced wounds in Wistar male rats. Indeed, sumac-treated rats at concentrations of 5 mg/mL and 10 mg/mL showed a significant wound healing at day 6 and 10 post-wounding associated with increased deposition of collagen, hydroxyproline, reduction of MMP-8, and MPO enzyme activity. The authors suggest that the improved healing process might be due to the anti-inflammatory activity of sumac fruit extract [61]. Macerated ethanol and ethanol-water fruit extracts of *Rhus coriaria* were shown to significantly downregulate in vitro, in HaCaT keratinocyte cells, the level of TNF-α- stimulated IL-8 through inhibition of the NFκB signaling pathway [123]. This study further confirms the potential therapeutic activity of *Rhus coriaria* in the treatment of skin inflammation. A large body of evidence points to the role of inflammation in cancer progression. Increased levels of inflammatory cytokines, such as IL-6, IL-8, and TNF-α, are known to promote migration, invasion and metastasis of various types of cancer [150] and inhibition of these pathways represent a promising approach in cancer treatment. Interestingly, *Rhus coriaria* ethanolic extract was shown to significantly downregulate IL-5, IL-8, and TNF-α in breast cancer cells [119]. In addition, *Rhus coriaria* fruit extract was shown to downregulate NFκB and STAT3 signaling, two major actors that play crucial roles in transmitting signals of inflammatory cytokines to the nucleus [119]. Hence, one possible mechanism through which *Rhus coriaria* inhibit breast cancer through its anti-inflammatory activity.

## 7. Toxicology Studies on *Rhus coriaria*

Since ancient times, herbal medicines have been used by humans not only due to their efficacy and safety but also due to their lower toxicity. However, recent studies pointed out that many of these medicinal herbs could exhibit some adverse effects [151]. Therefore, it is essential to evaluate the toxicological profile of any medicinal plant extract intended for medical use. *Rhus coriaria* was shown to be safe to be consumed by both humans and animals. Doğan and Çelik investigated the protective and therapeutic effect of *Rhus coriaria* extract on streptozotocin-induced diabetic rats. They have conducted toxicity test using three different dosages (250, 500, and 1000 mg/kg) of the plant extract. The results showed optimum tolerance and non-lethal oral uptake of *Rhus coriaria* lyophilized extract even at very high dose (1000 mg/kg) and there was no signs of toxicity and mortality after daily administration of the extract for 3 days [76]. Furthermore, another study showed that oral administration of 300 mg/kg hydroalcoholic *Rhus coriaria* seed extract has a favorable outcome in controlling some blood parameters in type 2 diabetic mice without causing any undesirable side effects [73]. Janbaz *et al.,* who demonstrated the antisecretory and antidiarrheal effect of *Rhus coriaria* crude extract in mice, reported that this extract was found to be safe even at dose of 5 g/kg [112]. Our group also showed that chick embryo treated with 150 µg/mL of ethanolic extract of this plant, a concentration shown to inhibit tumor growth and invasion of breast cancer cells, were perfectly healthy [119]. Taken together, these findings strongly indicate that this plant and its extracts are very safe, making them even more attractive for medicinal use or drug discovery approaches.

## 8. Beneficial Effect of *Rhus coriaria* in Food Industry

Recently, the use of spices as a source of natural antioxidants, preservatives, and fortifying agents in the food industries has gained much interest [152]. Gulmez et al. tested the ability of water extract of sumac fruit, used at concentration 8% (wt./vol.), to improve the bacteriological quality and refrigerated shelf life of broiler chicken meat [151]. The authors showed that the number of contaminating bacteria (psychotrophs, mesophilic aerobes, Enterobacteriaceae, coliforms, and fecal coliforms) grown on the meat, stored for 14 days at 25 °C, were reduced in sumac-treated compared to distilled water-treated broiler wings [153]. In addition, sumac-treated wings showed no sign of color fading or spoilation of the meat [153]. In another study, Arslan et al. tested the effect of several plant extracts—including sage, cinnamon, rosemary, sumac, clove, oregano, ginger, caraway, and thyme—on the shelf life of yayik butter [154]. Extract-treated butter was stored at 4 and 25 °C. Sumac fruit extract was shown to mildly increase the shelf life of yayik butter at both temperature [154].

Al-Marazeeq et al. tested the effect of water sumac fruit extract on the production of wheat pan bread. They showed that sumac extract improved in concentration-dependent manner the quality characteristics of the pan bread [155]. The authors attributed these improvements to the extractable organic acids, polyphenols, tannins, and anthocyanins from sumac. More recently, a similar study by Dziki et al. showed that wheat bread enriched with sumac flour extracted from sumac fruit also improved the quality of the bread evaluated by a decrease in the bread volume, lightness and yellowness of crumb and increase in the redness of the bread [156]. Also, the reduction of the amount of salt did not alter the quality of the sumac-enriched bread [156].

## 9. Conclusions and Future Perspectives

Despite the large number of activities identified in *Rhus coriaria* fruit powder and its safety profile, highlighted in this review, little attention was given toward the exploitation of these benefits in improving the human well-being. Sumac appears to be an excellent source of new compounds offering a broad spectrum of applications in different fields such as pharmaceutical, food, and textile industries. Because of its antioxidant, antifungal, and antibacterial activities, it may prove effective to test sumac as a natural preservative in food industry. More precisely, it would be beneficial to give more attention for the potential use of sumac powder as preservative in red meat and poultry meat. Also, much taught should be given toward the possible introduction of sumac extract as a supplement or condiment where sour taste is desired and obviously this will be better than the current use of natural or synthetic compounds such as citric acid. This later application could provide the consumer important phytochemicals beneficial for health with antioxidant and antiobesogenic potentials. Also, the rich composition in tannins, the large availability and low cost of sumac powder makes it interesting for use as cheap coloring agent by the textile industry. Last, and not the least, because of its demonstrated various pharmacological virtues (anticancer, anti-inflammatory, neuroprotective, antinociceptive, cardioprotective, and antidiabetic) the scientific community and pharmaceutical industry should give more attention toward the use of this plant as a source for novel bioactive compounds but also to conduct toxicological studies and clinical trials to develop better alternative natural pharmaceutical drugs.

## Figures and Tables

**Table 1:** Studies on the chemical composition of extracts from different parts of *Rhus coriaria* are illustrated in the table below.

Plant Part	Extract Type	Technique	Main Results	Ref.
**Fruits**	Petroleum ether extract	GC-MS	Fatty acids (oleic, linoleic, palmitic, and stearic acids) were present.	[12]
**Leaves**	Ethereal extract	Coloring method	Myricetin is the coloring agent of sumac. Also, gallic acid was identified in the extract.	[14]
**Fruits**	Aqueous Extract	HPLC	Organic acids (malic, citric, fumaric, and tartaric) were identified.	[16]
		GC	Fatty acid mainly oleic acid, linoleic acid, and palmitic acid were identified.	
**Fruits**	Methanolic extract	HPLC–DAD–ESI-MS/MS	The first extensive study of the phenolic and other phytochemical components of sumac fruit extract, where 211 compounds were identified.	[17]
**Leaves**	Ethyl acetate and methanol extracts	Paper chromatography	The flavonols quercetin, myricetin, and kaempferol were identified. Additionally, gallic acid, methyl gallate, m-digallic acid, and ellagic acid were identified as part of tannins.	[18]
**Leaves**	Ethyl acetate and methanol extracts	Paper chromatography, UV, and IR	Polyphenolic components of gallotannins were purely isolated.	[19]
**Leaves**	Ethyl acetate extract	Column chromatography	The presence of flavonoid glycosides was proved.	[20]
	Aqueous acetone extract	Partition solvent extraction	Methyl gallate was identified.	
**Leaves**	-	Liquid chromatography	The presence of monomeric flavonols (kaempferol, quercetin, and myricetin and rutin) and dimeric flavonoids (agathisflavone, amenthoflavone, hinokiflavone, and surnaflavone).	[21]
**Leaves**	Benzine extract	HPTLC	Polyisoprenoids were identified and they consisted of polyprenol homologs with 10–13 isoprene units. The dominant prenols were undecaprenols.	[22]
		GC-MS		
**Leaves**	Benzine extract	HPLC	Oxidized product of hexaprenol and the ester of tridecaprenol with linoleic acid were present in the fraction of the extract. The polyprenols content in the leaves was 2.5% of the air-dried leaf mass.	[23]
		ESI PI-MS		
**Leaves**	Aqueous extract	FIA	Ten gallotannins mono- to deca-galloyl glycosides of the class of hydrolysable tannins were identified. Coextracted flavonoid derivatives including myricetin, quercetin-3-O-rhamnoside, myricetin-3-O-glucoside, myricetin-3-O-glucuronide, and myricetin-3-O-rhamnoglucoside were also identified.	[24]
		HPLC		
		ESI–HR-MS/MS2		
**Fruits**	Phenolic fraction	Column chromatography	The presence of polyphenols: (flavonols, phenolic acids, hydrolysable tannins, and anthocyans) and organic acids (malic, citric, fumaric, and tartaric).	[25]
**Fruits**	Ethanolic extract	UV	Protocatechuic acid, isoquercitrin, and myricetin-3-O-α-*L*-rhamnoside were identified for the first time. Previously reported phenol acids and flavonoids, gallic acid, methyl gallate, kaempferol, and quercetin were identified.	[26]
		MS		
		IR		
		NMR		
**Fruits**	Methanolic extract	HPLC-MS	191 compounds were identified in sumac fruit including: 78 hydrolysable tannins, 59 flavonoid, 9 anthocyanins, and 40 other compounds such as butein.	[27]
**Fruits**	Methanolic extract	HPLC–MS	The presence of three different groups was identified. Anthocyanins: (cyanidin, peonidin, pelargonidin, petunidin, and delphinidin glucosides and coumarates), hydrolysable tannins: (pentagalloyl glucose) and phenolics: (gallic acid)	[28]
**Leaves and fruits**	Ethanolic and aqueous extracts	UPLC-PDA-ESI/MS	7-methyl-cyanidin 3-galactoside and gallic acid derivatives were identified. Anthocyanins were concentrated in sumac fruit.	[29]
**Fruits**	Ethanolic extract	LC-DAD-MS/MS	The presence of Phenolic compounds mainly gallic acid was proved.	[30]
		GC-MS	Identification of volatiles (β-pinene, octanal, limonene, nonanal, β-caryophyllene, and humulene) and fatty acids (oleic, palmitic, and linoleic acids).	
**Fruits**	Ethanolic extract	RRLC-DAD-ESI/MS	Gallicin, gallic acid, glucogallic acid, quercitrin, isohyperoside, myricetin glucuronide, tri-galloyl-hexoside, penta-galloyl-hexoside, myricetin rutinoside, dihydroxy-methyl xanthone, β-sitosterol-hexoside, α-tocopherol, linoleic acid.	[31]
**-**	Acetone extract	GC-MS	Alkaloid, tannin, saponins, and terpenoids and significant amounts of flavonoids and polyphenols were found in sumac.	[32]

**Table 2:** Studies on essential oil composition and the volatile profile of *Rhus coriaria* from different plant parts are illustrated in the table below.

Plant Part	Technique	Main Results	Ref.
**Fruits**	GC GC-MS	Over 120 constituents identified. Terpene hydrocarbons (i.e., α-pinene, β-caryophyllene and cembrene), oxygenated terpenes (i.e., *α*-terpineol, carvacrol and -caryophyllene alcohol), farnesyl acetone, hexahydrofarnesyl acetone and aliphatic aldehydes were the most abundant.	[34]
**Branches** **Leaves** **Fruits**	GC GC-MS	Sixty-three constituents identified with β-caryophyllene and cembrene being the most abundant.	[35]
		Sixty-three constituents identified with β-caryophyllene, sesquiterpene hydrocarbons (patchoulane) being the most abundant.	
		Eighty-five constituents identified with limonene, nonanal and (*Z*)-2-decenal being the most abundant.	
**Fruits**	GC GC-MS	The yield of the was 13.5% (*w*/*w*). 21 compounds representing 86.6% of the oils were identified among which β-caryophyllene (30.7%) and cembrene (21.4%) were the major constituents.	[36]
**Fruits** **Leaves**	HPLC TLC GLC	Fifty-four constituents identified from which β-caryophyllene, cembrene, (*E, E*) 2, 4-decadienal and α-terpineol were predominant.	[38]
		65 constituents with β-caryophyllene, caryophyllene oxide, cembrene and α–humulene were predominant.	
**Fruits**	HPLC GLC	Linoleic acid, tocopherols and sterols were predominant.	[39]
**Fruits**	GC-FID GC-MS HS-SPME	The main constituents were *p*-anisaldehyde, (*Z*)-2-heptenal, (*E*)-2-decenal, β-caryophllene and cembrene	[40]
**Stems** **Closed buds** **Open buds** **Leaves** **Flower buds** **Flowers** **Green fruits** **Green-red fruits** **Ripe fruits**	GC-FID GC-MS HS-SPME	α-pinene, (*E*)-β-ocimene, limonene, β-pinene (6.1%), myrcene (5.0%) (*Z*)-β-ocimene were predominant in the stems. β-caryophyllene and cembrene were the main constituents in leaves at all stages of maturation. α-pinene and tridecanoic acid were the major constituents in the flower.	[41]
**Fruits**	GC-FID GC-MS	Fifty-seven constituents were identified in the essential oil of sumac fruits collected from 14 different regions in Iran. (*E*)-Caryophyllene, n-nonanal (1.8–23.3%), cembrene (1.9–21.7%), α-pinene (0.0–19.7%), (2*E*,4*E*)-decadienal (2.4–16.5%) and nonanoic acid (0.0–15.8%) were identified as the main constituents	[42]

**Table 3:** Reported antioxidant and pharmacological activities of different parts of *Rhus coriaria* L.

Pharmacological Activities	Plant Part Used	Used Extract	Main Results	Ref.
**Antibacterial activity**	-	Acetone extract	*Rhus coriaria* was been reported to possess an antiparasitic activities against several piroplasm parasites, *Babesia bovis*, *B. bigemina*, *B. divergens, B. caballi*, and *Theileria equi* with an IC_50_ of 85 µg/mL, 55 µg/mL, 90 µg/mL, 85 µg/mL, and 78 µg/mL, respectively.	[32]
	Fruits	Aqueous extract	*Rhus coriaria* works as a stabilizing agent for the synthesis of copper nanoparticles. The synthesized CuNPs exhibited decent bacterial activity against *E. coli*, *Bacillus cereus*, *Staphylococcus aureus*, and *Pseudomonas aeruginosa*.	[59]
	Fruits	Hydroalcoholic extract	Sumac extract showed bactericidal effects against *Pseudomonas aeruginosa*, *Staphylococcus aureus*, *Acinetobacter baumannii*, and *Enterococcus faecalis*. It also inhibited the growth of both promastigotes and amastigotes with IC_50_ of 147 µg/mL and 233 µg/mL, respectively.	[60]
	Fruits	Lyophilized hydrophilic extract	A concentration of 5 and 10 mg/mL of sumac significantly inhibited the growth of *Staphylococcus aureus*, *Pseudomonas aeruginosa*, and methicillin-resistant *Staphylococcus aureus* (MRSA).	[61]
	Fruits	Essential oil extract	A concentration of 3 mg/mL significantly inhibited the growth of *Escherichia coli* and *Staphylococcus aureus* and a concentration of *15* mg/mL was required to inhibit the growth of *Pseudomonas aeruginosa* and *Bacillus subtilis.*	[62]
	Fruits	Ethanolic extract	*Rhus coriaria* inhibited the growth of *Salmonella enteric* and *Staphylococcus aureus* with a MIC of 0.78%.	[63]
	Fruits	Methanolic extract	*Rhus coriaria* extract at a concentration of 6 mg/mL inhibited Streptococcus *mutans* biofilm formation by 77%.	[64]
	Fruits	Methanol, acetone, alcohol and aqueous extracts	All sumac extracts at a concentration of 5 to 100 µg/mL exhibited the growth of *P. syringae* and *R. solanacearum*. *P. syringae* showed the most sensitivity to sumac, with MIC of 0.937 μg/mL while the MIC for *R. solanacearum* was 1.875 μg/mL.	[65]
	Epicarp of the fruits	Ethyl acetate extract	Sumac extract showed a strong inhibitory activity against *Staphylococcus aureus Escherichia coli.*	[66]
	Fruits	Aqueous extract	Sumac extract inhibited *Streptococcus mutans* biofilm formation with an MIC of 1.56 mg/mL.	[67]
	Fruits	Aqueous extract	*Rhus coriaria* had an antimicrobial activity against coliforms, *Listeria. Monocytogenes.*	[68]
	Fruits	Aqueous extract	Sumac extract inhibited the growth of *Streptococcus sanguinis*, *S. salivarius*, and *S. mutans* with a MIC of 1.562 mg/mL, of *Escherichia faecalis* with a MIC of 0.78 mg/mL and of *S. sobrinus* of a MIC of 0.39 mg/mL.	[69]
	Fruits	Aqueous extract	*Rhus coriaria* exhibited a substantial growth inhibition effect on *Staphylococcus aureus* in vitro and in vivo with a MIC of 0.025%	[70]
	Fruits	Water, methanolic and ethanolic extracts	*Rhus coriaria* ethanolic extract showed the highest growth inhibition activity against (Methicillin-resistant *Staphylococcus aureus (MRSA),* multi-drug resistant *Pseudomonas aeruginosa*, enterohemorrhagic *Escherichia coli (EHEC)*, *Proteus vulgaris*, and *Klebsiella pneumoniae* with a MIC of 1.25 mg/mL.	[71]
	Fruits	Aqueous extract	Sumac extract inhibited the following Gram (+) bacteria, *Bacillus cereus*, *B. megaterium*, *B. subtilis*, and *B. thuringiensis* with an MIC of 0.25–0.32%, *Staphylococcus aureus* with a MIC 0f 0.49% and *Listeria monocytogenes* with a MIC of 0.67%. It also efficiently inhibited Gram (-) bacteria, *Escherichia coli* Type I, *E. coli O157:H7, Proteus vulgaris and Hafnia alvei*, *Citrobacter freundii* with a MIC of 0.63%, 0.60%, 0.55%, 0.45% and 0.42%, respectively.	[72]
**Antidiabetic activity**	Seeds	Hydro-alcoholic extract	*Rhus coriaria* extract (300 mg/kg) significantly decrease the level of glucose and cholesterol and decreased in diabetic mice.	[73]
	Fruits	Powder	Supplementation of *Rhus coriaria* in the diet of type II diabetic women increased total antioxidant capacity, and significantly decreased insulin resistance index, blood glucose anthropometric measures (weight, body mass index)	[74]
	Fruits	Powder	Daily intake of 6 g of sumac powder decreased fasting serum insulin level and insulin resistance in patients with type II diabetes.	[75]
	Fruits	Lyophilized hydrophilic extract	Diabetic rats treated orally with Sumac extract (11, 250 and 500 mg/kg) for 21 days showed a significant decrease in the level of blood glucose, triglyceride, total cholesterol, high-density (HDL-c) and low-density (LDL-c) lipoprotein. Additionally, sumac extract caused a significant decreased the level of glycated hemoglobin (HbA1c) and α-glucosidase activity while it increased the level of insulin in serum of diabetic rats.	[76]
	Fruits	Aqueous extract	Sumac extract (ED50 of 11.5 ± 2.57 mg/mL) led to a significant decrease in the levels of blood glucose, LDL-c and alkaline phosphatase (ALP) activity in diabetic rats.	[77]
	Fruits	Powder	Type II Diabetic patients consuming 3.0 g sumac powder daily over 3 months showed a significant decrease in serum glucose and in the levels of HbA1-c and Aapo-B and a significant increase in the levels of HbA1-c and total antioxidant capacity.	[78]
	Seeds	Methanolic extract	Sumac (200 mg/kg and 400 mg/kg) administered orally daily for 5 weeks reduced the elevated levels of blood glucose, Hb1A-c and insulin in STZ-induced type II diabetes in rats. Sumac also significantly reduced the levels of blood glucose, total cholesterol, triglycerides, low density lipoprotein cholesterol (LDL-C) and very low-density lipoproteins cholesterol (VLDL-C), while it significantly increased the level of (HDL-C)	[79,80]
	Fruits	Ethanol extract	Alloxan-induced diabetic Wistar rats treated orally with sumac extract (200 mg/kg and 400 mg/kg) showed an efficient decrease in blood glucose only after one-hour treatment. Long term treatment (21 days) led to a significant reduction in the levels of postprandial blood glucose (PBG), LDL-C and significantly increased the level of HDL-C. It also increased the levels of superoxide dismutase (SOD) and catalase activities and inhibited the activities of maltase and sucrase.	[80]
	Fruits	Ethyl acetate extract	Ethyl acetate fraction of sumac fruits showed appreciable biological activity through α-amylase inhibition at an IC_50_ of 28.7 µg/mL highlighting potential hypoglycemic activity.	[81]
	Fruits	Aqueous extract	Sumac extract (100 and 200 μg/mL) moderately inhibited the growth of *Candida albicans* with a zone of inhibition > 8 mm. In addition, sumac (100 μg/mL) was able to significantly inhibit the adhesion of *Candida albicans* to the human HEp-2 epithelial cells in vitro.	[82]
**Antifungal activity**	Fruits	Aqueous extract	Sumac extract (100 and 200 μg/mL) moderately inhibited the growth of *Candida albicans* with a zone of inhibition > 8 mm. In addition, sumac (100 μg/mL) was able to significantly inhibit the adhesion of *Candida albicans* to the human HEp-2 epithelial cells in vitro.	[82]
	Seeds	Methanolic and Cream extract	Sumac methanolic extract (19 μg/mL) showed a significant antifungal activity against four dermatophytes (*Microsporum canis*, *M. gypseum*, *Trichophton equinum*, and *T. mentagrophyte*) responsible for dermatophytosis in human and animal. In addition, sumac cream (5%) applied daily for 10 days to Arabian horses with dermatophytosis led to a total healing from this infection after 28 days post-treatment.	[83]
	Fruits	Aqueous extract	*Rhus coriaria* fruit extracts at a MIC of 5 μg/mL inhibited the growth of *Colletotrichum acutatum* responsible for the anthracnose disease in tomato.	[84]
	Seeds	Ethanolic extract	Isolated new xanthone compounds from the seeds of *Rhus coriaria* possess antifungal activity against *Aspergillus flavus.*	[85]
	Leaf	Alcoholic extract	*Rhus coriaria* efficiently inhibited the growth of *Candida albicans* and *Aspergillus niger* with a MIC of 1 mg/mL and 0.5 mg/mL, respectively.	[86]
**Antioxidant activity**	Fruits	Methanolic extract	Sumac extract at concentration 1.0%, 3.0%, and 5.0% (wt./vol.) inhibited the formation of hydroperoxide and increase oxidative stability in peanut oil.	[13]
	Fruits	Lyophilized extracts	Sumac (100, 250, and 500 mg/kg) possesses antihemototoxic and antioxidant properties against STZ-induced diabetes mellitus rat model.	[30]
	Fruits	Ethanolic extract	Sumac extract (25 μg/mL) decreased UV-A-Induced ROS production in UV-A-treated human microvascular endothelial cells (HMEC-1). Also, sumac extract significantly reduced UV-A induced DNA damage in HMEC-1 cells.	[45]
	Fruits	Ethanolic extract	Sumac ethyl acetate fraction (IC_50_ 2.57 μg/mL) showed a strong antioxidant activity and exhibited the efficient protective effect against hydrogen peroxide-induced oxidative stress. Sumac crude ethanolic extract (1 and 3 μg/mL) protected human myoblast from H_2_O_2_-induced oxidative stress and restored their adhesion ability impaired by H_2_O_2_. Also, Sumac fraction protected zebrafish embryos from hydrogen peroxide-induced death in vivo.	[46]
	Fruits	Aqueous extract	Sumac extract (75 and 100 μg/mL) protected isolated rat hepatocyte against all oxidative stress induced by cumene hydroperoxide (CHP). Sumac extract protected rat hepatocytes against ROS generation, lipid peroxidation, glutathione depletion, mitochondrial membrane potential decrease, lysosomal membrane oxidative damage, and cellular proteolysis. In addition, sumac extract showed a strong H_2_O_2_ scavenging activity.	[56]
	Seeds	Hydroalcoholic extract	Sumac extract (200, 400, 800 mg/kg) administered orally or intraperitonially increased the total antioxidant capacity (TAC) in Wistar rats treated with sumac alone or in combination with morphine.	[87]
	Fruits	Aqueous extract	Sumac administered orally at a dose of 2 mL/kg per day prevented intestinal tissue damage in rat pups with induced necrotizing enterocolitis (NEC) through free radical scavenging activity and reduction of TNF-α and IL-6 levels.	[88]
	Fruits	Methanolic extract	Sumac (2 g/kg) showed a significant antigenotoxic activity against the genotoxic effect of urethane in rats.	[89]
	Fruits	Aqueous extract	*Rhus coriaria* alone and *Rhus coriaria*-synthesized nanoparticles showed a significant antioxidant activity using (ABTS^•+^) and (DPPH) assays.	[90]
	Leaf	Aqueous extract	*Rhus coriaria* water extract showed a high antioxidant capacity (Table 2). In particular, the antioxidant activity was 725.75 and 41.27 mg Trolox equivalent (TE)/g of water extract when the ABTS radical scavenging and ferric-reducing antioxidant power (FRAP) assays were used. Also, *Rhus coriaria*-fortified yogurt showed a significant increase in antioxidant activity in comparison with plain yogurt.	[91]
	-	Ethanolic extract	Sumac extract with IC_50_ of 29.89 μg/mL exhibited a strong antioxidant activity in DPPH radical scavenging assay. In addition, sumac significantly inhibited thiobarbituric acid reactive substances (TBARS) formation.	[92]
	Fruits	Acetone and ethanol extracts	Sumac acetone extract, rich in polyphenol content, showed a higher antioxidant activity compared to sumac ethanolic extract.	[93]
	-	Aqueous extract	Sumac extract (50, 100, and 200 mg/kg) decreased malondialdehyde, a marker of oxidative stress, while it increased catalase activities in the liver and kidney in alloxan-induced diabetic Wistar rats. Also, sumac extract was able to significantly reduce blood glucose in alloxan-induced diabetic rats.	[94]
	Fruits	Ethanolic extract	Orogastrically administered sumac (20 mg/kg/day) to Wistar rats with Ligature-induced periodontitis reduced alveolar bone loss by affecting RANKL/OPG balance, total oxidant status and oxidative stress index levels in the treated rats.	[95]
	Fruits	Powder	Daily consumption of sumac extract for 90 days increased the total antioxidant status (TAS) and albumins while it decreased cholesterol levels in in adult male rabbits.	[96]
	Fruits	Aqueous extract	Sumac showed a strong antioxidant and free radical scavenging activity. Sumac water extract of scavenged radicals effectively with EC_50_ of 36.4 μg/mL for DPPH free radical and 44.7 μg/mL for DMPD cation radical.	[97]
	Leaf	Ethanolic extract	Sumac extract (10, 100, and 200 μg/mL) reduced the levels of ROS, NO, and PGE_2_ production induced by IL-1β in Human articular chondrocyte. Furthermore, sumac alleviated the inhibitory effect of IL-1β on the synthesis of glycosaminoglycans in the human chondrocytes.	[98]
	Fruits	Methanolic extract	Sumac methanolic extract showed considerable antioxidant scavenging activity against free superoxide radicals (IC_50_ 282.92 μg/mL), hydroxyl radicals (IC_50_ 3.85 g/mL) and lipid peroxidation (IC_50_ 1.2 g/mL) in vitro.	[99]
	Fruits	Methanolic extract	*Rhus coriaria* extract, acts as an uncompetitive inhibitor of xanthine oxidase and scavenger of superoxide radical in vitro with IC_50_ values of 172.5 μg/mL and 232 μg/mL, respectively.	[100]
**Antinociceptive activity**	Leaf	Hydro-alcoholic extract	*Rhus coriaria* showed a considerable antinociceptive activity in Wistar rats. Sumac extract (300 mg/kg) injected intraperitoneally to Wistar rats caused a significant reduction in writhing number caused by acetic acid, an increase in tail-flick latency, and decreased pain score in both acute and chronic phases in the formalin test.	[101]
**Cardioprotective and antidyslipidemia** **activity**	Fruits	Ethanolic extract	*Rhus coriaria* extract (0.3–1.0 mg/mL) induced a concentration-dependent endothelium-dependent vasorelaxation of rat aorta. The sumac-dependent vasorelaxation was achieved via stimulation of multiple transducers namely PI3-K/Akt, endothelial nitric oxide synthase (eNOS), NO, guanylyl cyclase, cGMP, and PKG.	[102]
	Fruits	Powder	The consumption of sumac (500 mg, twice daily) for 4 weeks led to a significant decrease in the body mass index (0.21 ± 0.075 kg/m^2^), systolic blood pressure (1.87 ± 0.83 mm Hg), diastolic blood pressure (1.32 ± 0.46 mm Hg), and total cholesterol (14.42 ± 4.95 mmol/L) while significantly increased the flow-mediated dilation (−0.23 ± 0.065%) in adult patients with hyperlipidemia.	[103]
	Fruits	Powder	*Rhus coriaria* supplementation, at a dose of 1 g/day for 6 weeks, in patients with hyperlipidemia showed significant increases in HDL-C and Apo-A1 levels.	[102,104]
	Fruits	Powder	The consumption of sumac powder (500 mg, three times daily) for 4 weeks led to a significant reduction of total cholesterol, LDL-C, and triglyceride in obese adolescents with dyslipidemia.	[105]
	Fruits	Methanolic extract	The levels of total cholesterol and triglyceride were significantly reduced in hypercholesterolemic rats treated with sumac extract (100 and 200 mg/kg/day) for 15 days. Also, the level of serum of two injury marker exams, aspartate aminotransferase and alanine aminotransferase, were reverted to near normal in rats fed with high cholesterol diet.	[106]
	Leaf	Hydro-alcoholic extract	Hydrolysable tannins isolated from *Rhus coriaria* leaves induced a dose-dependent normalization of coronary perfusion pressure, reduced left ventricular contracture during ischemia, and improved left ventricular developed pressure and the maximum rate of rise and fall of left ventricular pressure at reperfusion in male rabbits. The cardiovascular protective effect of sumac could be attributed to COX pathway activation, TNF-α inhibition, eNOS activation, and free radical and ROS scavenging.	[107]
	-	Grounded dry sumac	Tannin extracted from *Rhus coriaria* reduced by 62% the migration of vascular smooth muscle cells (VSMC).	[108]
**Neuroprotective activity**	Fruits	Ethanolic extract	Mice treated with 400 mg/kg of sumac extract, after optic nerve injury, exhibited 84.87% inhibition of ischemia, determined by fluorescence molecular tomography (FMT) imaging.	[109]
**Dental protection** **activity**	Seeds	Aqueous extract	Sumac extract (10% wt./vol.) caused a significant reduction in the endogenous matrix metalloproteinase (MMP-2, 8 and 9) activity of demineralized dentin matrix. Also, sumac was able to increase the intra and interfibrillar crosslinking density of dentin collagen matrix.	[107,110]
**Antidiarrheal activity (Gut protective effect)**	Fruits	Methanolic Extract	Sumac extract affect metabolic pathways of human gut microbiota of human. Treatment of a consortium of six microorganisms’ representative of intestinal human microbiota with sumac extract (0.5 and 5 mg/mL) led to a decrease in the levels of amino acids and nitrogenous compounds in the bacteria cultures.	[111]
	Fruits	Aqueous extract	*Rhus coriaria* (100 and 300 mg/kg) demonstrated antisecretory, antidiarrheal effects against castor oil-induced fluid accumulation and diarrhea. Sumac extract exhibited an antispasmodic activity in isolated rabbit jejunum. The extract reduced the high K^+^-induced spastic contractions with EC_50_ of 0.35 mg/mL and exerted a Ca^++^ antagonist in rabbit jejunum.	[112]
**Effect on laying hens and eggs and broiler chickens**	Fruits	Powder	Broilers, for which sumac powder (1% and 3% of total diet) was included in the diet, demonstrated an improved immune system against Newcastle Disease and influenza. A reduced fat content was also observed in sumac-fed broilers.	[113,114]
	Seeds	Powder	Addition of sumac (10–30 g/kg) in the diet of laying hens reduced the levels of yolk and blood cholesterol. Sumac was also shown to lower crude fat content.	[115,116]
**Anticancer activity**	Fruits	Ethanolic extract	*Rhus coriaria* at non-cytotoxic concentration (31.25, 62.5, and 125 μg/mL) inhibited the migration of uterus cervix cancer (HeLa) cells.	[31]
	Fruits	Aqueous extract	Silver nanoparticles made from sumac extract (AgSu/NP) exhibited cytotoxic (IC_50_ of ~10 µmol/48 h) and pro-apoptotic effect on breast cancer (MCF-7) cells.	[117]
	Fruits	Ethanolic extract	*Rhus coriaria* showed anti-colon cancer activity via stimulation of proteasome activity and induction of autophagic and apoptotic cell death in HT-29 (IC_50_ at 24 and 48 h were 518 and 346 µg/mL) and Caco-2 (IC_50_ at 24 and 48 h were 384 and 316 µg/mL) cell lines.	[118]
	Fruits	Ethanolic extract	*Rhus coriaria* exhibited anti-breast cancer activity by suppressing metastasis, angiogenesis, and tumor growth via inhibition of via inhibition of STAT3, NFκB, and nitric oxide pathways.	[119]
		Oleoresin extract	*Rhus coriaria* inhibited angiogenesis and showed cytotoxic effect (IC_50_ of 9.1 µg/mL) against retinoblastoma (Y79) cancer cells.	[120]
	Fruits	Ethanolic extract	*Rhus coriaria* inhibited cell viability of MDA-MB-231 (IC_50_ of 305 µg/mL at 48 H), T47D (IC_50_ of 261 µg/mL at 48 H) and MCF-7 (IC_50_ of 510 µg/mL at 48 H) breast cancer cells. In addition, Sumac extract induced senescence and autophagy in triple negative breast cancer cells via the activation of p38 and ERK1/2 pathways.	[121]
**Anti-inflammation** **activity**	Fruits	Alcoholic extract	*Rhus coriaria* showed anti-inflammatory effects by reducing IL-1β, IL-18 expression in lipopolysaccharide-stimulated synoviocytes.	[122]
	Fruits	Ethanolic extract	*Rhus coriaria* L. showed a potential to treat skin inflammatory conditions in HaCaT cells by inhibiting the release of pro-inflammatory mediator IL-8.	[123]
**Wound healing** **activity**	Fruits	Lyophilized hydrophilic extract	Sumac fruit extract (5 mg/mL and 10 mg/mL ) accelerates the healing of induced wounds in Wistar male rats.	[61]
	Fruits	Ethanolic extract	Sumac extract (100 and 200 mg/kg) promoted a fast and efficient wound closure in wounded male Sprague Dawley rats.	[124]
**Other biological** **activities**	Fruits	-	*Rhus coriaria* possesses a potential allelopathic activity. It reduced lettuce radicle and hypocotyl elongation to 7.4% and 33.1% of control, respectively, in sandwich method bioassay.	[125]
	Fruits	Grounded and packed into dark sachets	Clinical trials showed that *Rhus coriaria* in combination with *Bunium persicum B.* reduced chemotherapy induced nausea and vomiting (CINV) phase in breast cancer patient.	[126]
	Fruits	Aqueous extract “Juice”	Oral intake of sumac juice showed a beneficial impact on muscle performance among athletes and reduced pain during exercise.	[127]
	-	Methanolic extract	*Rhus coriaria* showed prevention effect from gastrointestinal diseases via inhibiting urease enzyme activity.	[128]
